# Incorporation of doxorubicin into plant-derived nanovesicles: process monitoring and activity assessment

**DOI:** 10.1080/10717544.2024.2439272

**Published:** 2024-12-11

**Authors:** Aleksandra Steć, Monika Targońska, Shishir Jaikishan, Rui Chen, Piotr Mucha, Grzegorz S. Czyrski, Jacek Jasiecki, Agata Płoska, Andrea Heinz, Susanne K. Wiedmer, Leszek Kalinowski, Krzysztof Waleron, Bartosz Wielgomas, Szymon Dziomba

**Affiliations:** a Department of Toxicology, Faculty of Pharmacy, Medical University of Gdansk, Gdansk, Poland; b Department of Biology and Medical Genetics, Medical University of Gdansk, Gdansk, Poland; c Department of Chemistry, University of Helsinki, Helsinki, Finland; d Faculty of Chemistry, Laboratory of Chemistry of Biologically Active Compounds, University of Gdansk, Gdansk, Poland; e Department of Pharmacy, LEO Foundation Center for Cutaneous Drug Delivery, University of Copenhagen, Copenhagen, Denmark; f Department of Pharmaceutical Microbiology, Faculty of Pharmacy, Medical University of Gdansk, Gdansk, Poland; g Department of Medical Laboratory Diagnostics—Fahrenheit Biobank BBMRI.pl, Faculty of Pharmacy, Medical University of Gdansk, Gdansk, Poland; h Department of Mechanics of Materials and Structures, BioTechMed Centre, Gdansk University of Technology, Gdansk, Poland

**Keywords:** Capillary electrophoresis, drug incorporation, exosome-like vesicles, extracellular vesicles, nanocarriers, nanoplasmonic sensing

## Abstract

Extracellular vesicles (EVs) are an experimental class of drug carriers. Alternative sources of EVs are currently being explored to overcome limitations related to their manufacturing from mesenchymal stem cells. In this work, *Citrus limon-*derived EVs were tested as carriers for the widely used chemotherapeutic drug – doxorubicin (DOX). Capillary electrophoresis (CE) and nanoplasmonic sensing (NPS) were developed for the quality control of DOX–EV preparations. It was found that the CE method enables simultaneous detection of free and incorporated DOX and allows assessing the stability of the preparations and the drug leakage. NPS, on the other hand, demonstrated that DOX is accumulated in the interfacial region of the carrier. The activity of DOX-loaded EVs was tested on HeLa (cervical cancer cells) and HEK293T (human embryonic kidney cells) cell lines. It was found that DOX incorporation into plant-derived EVs virtually does not affect the drug’s cytotoxicity to HeLa cells but significantly decreases DOX activity against HEK293T cell line.

## Graphical Abstract

**Figure:**
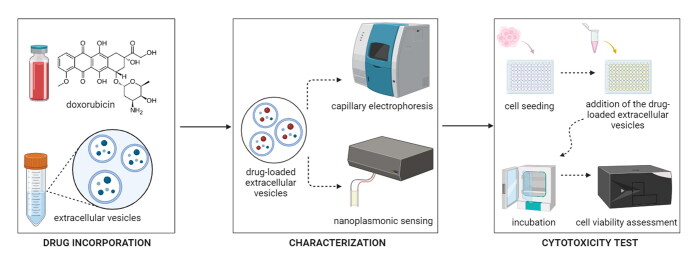

## 1. Introduction

Extracellular vesicles (EVs) are biological, nano- and microparticles released by living cells (Van Niel et al., 2018). Due to their ability to transport various biologically active substances, EVs are currently considered a novel class of biologicals. Their potential in regenerative medicine and targeted drug delivery is under investigation (Du et al., 2023).

Doxorubicin (DOX) is a frequently prescribed anticancer drug (Chatterjee et al., 2010). The most accepted mechanism of action of DOX is through direct intercalation between the base pairs in the DNA helix, leading to inhibition of DNA replication and damage. Interaction of DOX with membranes is also inevitable as the drug must pass through the cell membrane and, finally, the nuclear membrane if the drug is to interact with DNA (de Wolf et al., 1993; Alves et al., 2017; Matyszewska et al., 2021; Ceballos et al., 2023). DOX reportedly forms oxygen free radicals, leading to cytotoxicity next to lipid peroxidation of membrane lipids. This mechanism is responsible for numerous adverse effects observed during DOX treatment, including cardiomyopathy (Deng et al., 2007; Chatterjee et al., 2010). The risk of cardiotoxicity is significantly reduced when liposomal formulations of DOX are administered instead of infusion of free drug (Xing et al., 2015). A few polyethylene glycol-modified (PEGylated) and non-PEGylated liposomal formulations of DOX are currently available and approved by the Food and Drugs Administration (FDA) agency and the European Medicines Agency (EMA). Other concepts of DOX carriers, currently under clinical trials, include polymeric micelles, polymeric nanoparticles, and polymer–drug conjugates (Lee et al., 2023).

The utilization of EVs as delivery vehicles is one of the most recent concepts and is gaining importance due to their substantial benefits over traditional drug delivery methods (Farhat et al., 2022; Li et al., 2022; Patras et al., 2022; Mukhopadhya et al., 2023). EVs are capable of targeted drug delivery due to the possibility of systemic cell-to-cell communication. Vesicles are also considered to be biocompatible, low immunogenic, and capable of penetrating biological barriers (Du et al., 2023). EVs can be chemically modified or bioengineered to improve their pharmacokinetics (Herrmann et al., 2021). In the case of DOX, a decreased risk of cardiomyopathy has already been proven with modified human embryonic kidney HEK-293 cell line-derived vesicles used as carriers (Martins-Marques et al., 2016). The treatment efficiency of grapefruit-derived EVs, modified with heparin-based nanoparticles and loaded with DOX, has recently been demonstrated on a mice glioma model (Niu et al., 2021). The modification enabled targeted drug delivery to animal brains and led to a fourfold increased capacity of drug loading as compared to unmodified vesicles. PEGylation of cell culture-derived vesicles was also beneficial in *in vivo* tests (Patras et al., 2022). PEGylated vesicles, loaded with DOX, featured superior antitumor activity compared to free DOX and its liposomal formulation administration. Bovine milk-derived EVs, decorated with hyaluronan, were able to selectively deliver DOX to tumor cells overexpressing CD44 receptors under *in vitro* conditions (Li et al., 2020). Other publications assessed various types of EVs (including eukaryotic cell lines (Schindler et al., 2019; Farhat et al., 2022; Li et al., 2022; Chen et al., 2023; Mukhopadhya et al., 2023) and bovine milk (Chen et al., 2021; Mukhopadhya et al., 2023)) as DOX carriers, presenting a modulatory effect of the formulations on the cytotoxicity of the drug on selected cancer cell lines.

The optimization of drug loading conditions and the quality control of developed formulations containing EVs as drug carriers is demanding. The methodologies typically used for the assessment of DOX incorporation efficiency are based on spectrophotometric measurement of the EV preparation after free drug removal using size exclusion chromatography (SEC) (Chen et al., 2021), ultracentrifugation (Martins-Marques et al., 2016; Niu et al., 2021; Li et al., 2022; Chen et al., 2023), or filtration techniques (Mukhopadhya et al., 2023). Similar approaches were implemented for the assessment of drug leakage from the carriers. Chen et al. measured the fluorescence of the acceptor phase during dialysis of DOX-loaded EVs (Chen et al., 2021). Li et al. performed spectrophotometric measurements of the drug released during incubation of the formulation in ultrafiltration (UF) concentrators, centrifuged at specific time points (Li et al., 2022). However, in the former case, the assay was performed in a two-compartment system (which is not typical for pharmaceutics’ stability assessment), and in both cases, semi-permeable membranes were used, which might affect the assay due to the drug and/or EV adsorption to the membrane surface. Furthermore, the implementation of additional procedures such as chromatographic separation, ultracentrifugation, dialysis, or filtration, significantly increases the complexity of the assay and negatively affects the duration, cost, and accuracy of the measurement. An interesting alternative approach has recently been proposed by Yan’s group. Using nano-flow cytometry, the group reported decreased DOX encapsulation during formulation storage (Chen et al., 2023). However, the analysis was based on a decrease in the fluorescence of the particle, and the concentration of free DOX was not measured.

This work focuses on the characterization of EVs loaded with DOX. We employed capillary electrophoresis (CE) for investigating DOX incorporation into plant-derived EVs (*Citrus limon*) and simultaneously the formulation stability and drug leakage processes were assessed. We also demonstrate the utility of nanoplasmonic sensing (NPS) for the analysis of drug-loaded EVs. The activity of the obtained formulations was tested on HeLa and HEK293T cell lines and the results are discussed in the context of further research.

## 2. Materials and methods

### 2.1. Chemicals and cell lines

Glycine (Gly), BIS–Tris propane (BTP), bovine serum albumin (BSA), phosphate-buffered saline (PBS), sodium dodecyl sulfate (SDS), 2-amino-2-hydroxymethyl-propane-1,3-diol (Tris), 4-(2-hydroxyethyl)-1-piperazineethanesulfonic acid (HEPES), and DOX hydrochloride were obtained from Merck (Steinheim, Germany). Sodium hydroxide (NaOH) pellets were purchased from J.T. Baker Chemicals (Center Valley, PA). Calcium chloride (CaCl_2_) was purchased from VWR International (Espoo, Finland). All other inorganic and organic chemicals were of the highest purity available. All chemicals were of analytical grade. In experiments, deionized water (resistivity of 18.2 MΩ cm) was used.

HEK293T (CVCL_0063; cat. no. CRL-3216) and HeLa (CVCL_0030; cat. no. CRM-CCL-2) cell lines were obtained from ATCC (Manassas, VA). Cell Counting Kit-8 (CCK-8), based on solution of WST-8 [2-(2-methoxy-4-nitrophenyl)-3-(4-nitrophenyl)-5-(2,4-disulfophenyl)-2H-tetrazolium, monosodium salt] was purchased from Merck (Steinheim, Germany). Minimum essential medium (MEM), fetal bovine serum (FBS), and PenStrep were purchased from Thermo Fisher Scientific (Waltham, MA).

### 2.2. Isolation of plant EVs

EVs were isolated from *Citrus limon* juice using a method developed by our group (Steć, Chodkowska, Kasprzyk-Pochopień, et al., 2023). A combination of UF and SEC was used for the isolation. Lemons were purchased in a local grocery store and washed with lukewarm water. The juice was mechanically squeezed (about 40 mL from a single fruit) and centrifuged for 30 min at low speed (3000 RCF) in a Sorvall 16R centrifuge (Thermo Fisher Scientific, Waltham, MA) using a swing-out bucket. The supernatant was collected and subsequently centrifuged at 10,000 RCF and 25,000 RCF using a fixed-angel rotor. After each centrifugation process, the pellet was discarded. Aliquot of 10 mL of the finally obtained supernatant was preconcentrated down to 0.5 mL using a Vivaspin 15 concentrator (100 kDa; polyethersulfone membrane; Sartorius, Göttingen, Germany). The whole retentate was then fractionated with a PD MiniTrap G-25 column (Cytiva, Marlborough, MA) using a PBS solution. Fractions 2 and 3 (0.5 mL each) were combined and concentrated with Amicon Ultra 0.5 (Merck, Steinheim, Germany) to a final volume of 0.5 mL. Isolated vesicles were stored at 4 °C until further use.

### 2.3. Incorporation of DOX into plant EVs

An aqueous DOX solution was mixed with EV isolate (the isolation procedure was described in Section 2.2) in a volumetric ratio of 1:9 and incubated at 37 °C for 24 h. Afterwards, free DOX was removed using a PD MiniTrap G-25 column (Cytiva, Marlborough, MA). 0.5 mL of the DOX and EVs mixture was transferred into the column and eluted with PBS solution. Fractions 2 and 3 (0.5 mL each) were combined and concentrated with Amicon Ultra 0.5 (Merck, Steinheim, Germany) to a final volume of 300–500 µL.

### 2.4. Total DOX quantitation

Quantification of the total DOX content in the obtained preparation was performed using an Infinite 200 plate reader (Tecan, Mannedorf, Switzerland). DOX-loaded EV preparations and DOX standard solutions were mixed with 6% (w/w) SDS solution in a 9:1 volumetric ratio to disrupt the vesicle integrity. Before the measurement, 20 µL of this mixture was diluted with 100 µL of 30 mM phosphate buffer (pH 2.1). Fluorescence was measured at 488 nm (excitation wavelength) and 630 nm (emission wavelength). The drug content was calculated based on the calibration curve obtained with DOX standard solutions in PBS.

### 2.5. Bicinchoninic acid (BCA) assay

Protein content measurement was performed with a Pierce BCA kit (Thermo Fisher Scientific, Waltham, MA) according to the vendor’s recommendations. Samples and standard solutions were mixed with 6% (w/w) SDS solution in a 9:1 volumetric ratio to disrupt the vesicle integrity. Ten microliters of these mixtures were transferred to a 96-well plate. Two hundred microliters of BCA reagents mixture was added to each well and the plates were incubated at 37 °C for 30 min. The spectrophotometric measurement was performed using an Infinite 200 plate reader (Tecan, Mannedorf, Switzerland) at 562 nm. The total protein content was determined based on the calibration curve constructed with BSA.

### 2.6. Nanoparticles tracking analysis (NTA)

Analyses were performed using a Nanosight NS300 instrument (Malvern Instruments, Malvern, UK) with NTA software (version 3.2 Dev Build 3.2.16, Malvern Instruments, Malvern, UK). Samples were diluted to the recommended concentration range (10^7^ to 10^9^ particles mL^−1^) using PBS solution (pH 7.4). Measurements were done in duplicate using a 405 nm laser and an sCMOS camera. During a single analysis, five videos were recorded. The camera level was set to 15 or 16, with a focus of 180–220 at 25 °C.

### 2.7. Capillary electrophoresis

All CE experiments were conducted with a PACE MDQ plus system (Sciex, Framingham, MA). Two types of detectors were used in the presented experiments: a diode array detector (DAD) or a laser-induced fluorescence (LIF) detector. DAD was used for quality control of EV isolates and LIF was used for the monitoring the fluorescence of DOX at 488 nm (excitation) and 630 nm (emission).

The CE methodology was partially described in Dziomba et al. (2021) and Steć, Chodkowska, Kasprzyk-Pochopień, et al. (2023). Electrophoresis was conducted using uncoated fused silica capillaries (50 µm i.d. × 363 µm o.d. × 30.2 cm of total length) at a positive voltage of 10 kV. For the quality control of EVs isolated from *Citrus limon* juice, the background electrolyte (BGE) was composed of 50 mM BTP and 75 mM Gly (pH 9.5). The analysis of DOX standards and DOX-loaded plant-derived EVs was performed with a BGE composed of 50 mM Tris and 200 mM HEPES (pH 6.9) using LIF detection.

At the start of each working day, the capillary was conditioned with 0.1 M NaOH for 10 min, rinsed with water for 10 min, and then conditioned with BGE for 10 min. At the end of the working day, the capillary was subsequently rinsed with 0.1 M solution of NaOH and water for 10 min each, and the capillary outlets were stored in water until further use.

Before each analysis, the capillary was conditioned with 0.1 M solution of NaOH for 2 min, water for 1 min, and BGE for 5 min. A water-dipping procedure was then performed to prevent sample contamination, followed by sample injection for 5 s at 3.45 kPa. A post-injection of BGE for 5 s at 3.45 kPa was carried out before the voltage was applied. A ramp time of 0.5 min was used to reach the constant voltage. All rinsing and conditioning steps were performed under a pressure of 137.9 kPa.

### 2.8. Nanoplasmonic sensing

Localized surface plasmon resonance-based NPS measurements were performed on a nanodisk in optical transmission mode using an Insplorion X2 XNano instrument (Insplorion AB, Gothenburg, Sweden). The sensing surface employed for this purpose was a silicon dioxide sensor chip (Insplorion AB, Gothenburg, Sweden). Prior to the experiment, the sensor was subjected to ultrasonic cleaning (5 min) in ethanol. After drying under nitrogen, a 5 min pretreatment was performed on the sensor surface using a UV Ozone Cleaner (UVC-1014 NanoBioAnalytics, Berlin, Germany) to remove organic contaminants. To prevent bubble interference during the measurements, all buffer solutions were degassed for 30 min in an Elmasonic P 30 H ultrasonic cleaning unit (80 Hz, Elma, Singen, Germany). All experiments were repeated twice under a continuous flow rate of 60 μL min^−1^, controlled by a Reglo-CPF digital peristaltic pump (Ismatec, Wertheim, Germany). The system temperature was maintained at 25 °C using the Insplorion XNano temperature control unit. To ensure consistency, all measurements were performed using sensors from the same batch, minimizing inter-batch variations. The overall injection procedure involved sequential pretreatment with Milli-Q water, 0.1 M NaOH, Milli-Q water, 10 mM HEPES buffer, and 10 mM HEPES buffer containing 5 mM CaCl_2_ (10 mM Ca–HEPES). Subsequently, samples of EVs (the isolation procedure is described in Section 2.2) were injected, followed by sequential rinsing of the sensor and tubing with 10 mM Ca–HEPES buffer and Milli-Q water for 3 min each.

### 2.9. Electrophoretic light scattering (ELS)

Zeta potential measurements were performed with Zetasizer Nano ZS (Malvern Instruments, Malvern, UK) using U-curved disposable cuvettes (DTS1070; Malvern Instruments, Malvern, UK). The refractive index of the material and water were set to 1.45 and 1.33, respectively. The viscosity of the dispersant was 0.8872 cP (mPa s), and material absorption was 0.001. Native and DOX-loaded EVs, prepared as described in Sections 2.2 and 2.3, were 20-fold diluted with deionized water before the measurement. The Hückel model was used for zeta potential calculations. Each sample was analyzed in triplicate.

### 2.10. Cell culture of HEK293T and HeLa

HEK293T and HeLa cells were maintained in MEM supplemented with 10% (v/v) FBS, 100 units mL^−1^ penicillin, and 100 μg mL^−1^ streptomycin. Cells were grown at 37 °C under 5% (v/v) CO_2_. The cells were passaged at 90% confluence every three days.

### 2.11. Cytotoxicity assays

Cells were seeded at 20% confluence (6.4 × 10^3^ cells) in the 96-well plate in 100 μL of the medium. After 24 h, 10 μL of the DOX-loaded EVs were added to the medium. Additionally, to determine the IC_50_ of DOX, the DOX solution in PBS was added to the wells at increasing concentrations. The cells were incubated for 48 h. To assess the toxicity profile of the formulations, CCK-8 reagent was added to the medium at a volumetric ratio of 1:10, and then the cells were incubated for 4 h. Next, the absorbance was measured at 450 nm using the TECAN Infinite M200PRO, and the percentage of living cells was determined.

### 2.12. Imaging of cells using confocal microscopy

6.4 × 10^3^ cells per well were cultured in 100 μL of medium supplemented with the addition of DOX solution in PBS (decreasing concentrations) or DOX-loaded EVs (Section 2.3) in 96 Well Cell Carrier™-96 ultra plates (PerkinElmer, Waltham, MA) for 48 h. Next, the medium was replaced with fresh cultured medium. The images were obtained using Opera Phenix High Content Screening System and Harmony 4.8 software (PerkinElmer, Waltham, MA) with a 63x water immersion objective (NA 1.15) with sustaining life conditions (37 °C, 5% (v/v) CO_2_). The DOX signal was visualized with a 561 nm bandpass excitation filter and a 570–630 nm bandpass emission filter. Images were processed with Harmony 4.8 software (PerkinElmer, Waltham, MA). The relative fluorescence intensities of DOX were calculated using the ImageJ software (NIH, Bethesda, MD).

### 2.13. Cryogenic transmission electron microscopy (cryo-TEM)

The morphology of plant EVs (the isolation procedure is described in Section 2.2) was investigated by cryo-TEM. For this purpose, 3 µL of the isolate was blotted onto a discharged Lacey formvar/silicon monoxide 300 mesh copper grid (Ted Pella Inc., Redding, CA) with a blotting force of 2, a blotting time of 3 s, and a temperature of 4 °C using a Vitrobot Mark IV (Thermo Fisher, Waltham, MA). The grid was then plunged into liquid ethane to vitrify it. Images of the EVs were captured at various magnifications using a FEI Tecnai G2 20 TWIN transmission electron microscope (Hillsboro, OR), which was operated at a voltage of 200 kV in a low-dose setup, and a FEI High-Sensitive Eagle camera.

## 3. Results

### 3.1. Isolation of plant EVs

**Figure 1.**
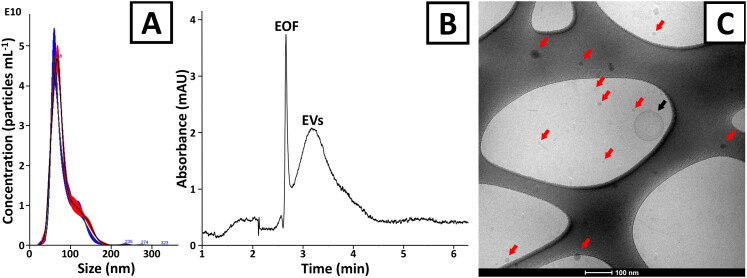
The characterization of EV isolates obtained from *Citrus limon* juice. (A) The size distribution of (red trace) native and (blue trace) DOX-loaded vesicles (NTA). (B) CE analysis of EV isolate. The analysis was performed with UV detection (230 nm) and the BGE composed of 50 mM BTP/75 mM Gly (pH 9.5). (C) Cryo-TEM image of native vesicles. Black and red arrows indicate microvesicles and exosome-like plant vesicles, respectively. The bar at the bottom corresponds to 100 nm. An unprocessed cryo-TEM image and a zoom out image are provided in Figures S1 and S2, respectively.

Plant EVs were isolated according to the methodology described in Section 2.2. The obtained isolates were characterized with NTA (Figure 1(A)). Particle size distribution was similar to previously reported size distributions and ranged from about 30 to 200 nm (Steć, Chodkowska, Kasprzyk-Pochopień, et al., 2023). The mean, mode, and median sizes (±SD) of the vesicles were: 88 ± 4 nm, 68 ± 5 nm, and 79 ± 4 nm, respectively (calculated based on the analyses of three independently obtained isolates, *n* = 3). The total isolation yield obtained from 10 mL of a raw plant material, estimated based on BCA and NTA measurements, was 210 ± 37 µg and 5.1 × 10^11^ ± 2.1 × 10^11^ particles, respectively. The purity of isolates was estimated using CE (Steć, Chodkowska, Kasprzyk-Pochopień, et al., 2023; Steć, Targońska, Karkosińska, et al., 2023). Relatively symmetrical and poorly efficient (number of theoretical plates were <20,000 m^−1^) signals, characteristic of EVs, were observed (Figure 1(B)) (Morani et al., 2020; Piotrowska et al., 2020; Steć, Chodkowska, Kasprzyk-Pochopień, et al., 2023). No additional signals indicating the presence of impurities were detected. The particle number and protein content ratio (2.5 × 10^9^ particles µg protein^−1^) were also comparable with previous reports (Steć, Chodkowska, Kasprzyk-Pochopień, et al., 2023).

In our previous work, proteomics by mass spectrometry indicated the presence of tetraspanin-8 (TET-8) and syntaxin-121 (PEN1) in the isolated EVs (Steć, Chodkowska, Kasprzyk-Pochopień, et al., 2023). These proteins have been identified as proteomic markers of plant exosomes and microvesicles, respectively (Cai et al., 2021). The application of cryo-TEM in the current paper enabled us to clearly distinguish both types of vesicles. Microvesicles were observed to be relatively big (usually >100 nm), spherical structures with clearly distinguishable cytosolic membranes (Figure 1(C) and Figure S1). Exosome-like plant-derived EVs were significantly smaller (≪100 nm) and featured a greater electron density (‘small dark spots’ visible in Figure 1(C)). The latter ones were much more abundant than the microvesicles (Figure S2). A rough estimation of the microscopic images led us to the conclusion that ≫90% of all isolated particles were identified as exosome-like plant-derived EVs.

### 3.2. DOX-loading into plant-derived EVs

**Figure 2.**
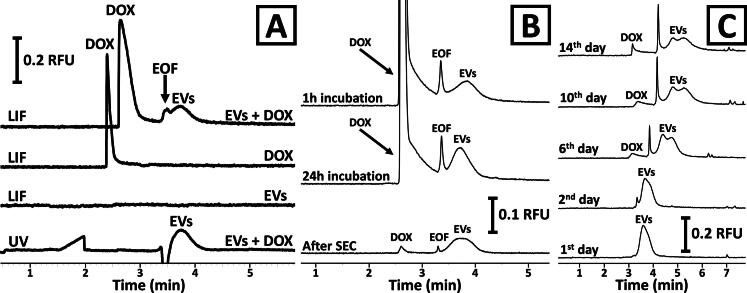
CE of DOX loading into plant-derived EVs. (A) Analyses of samples containing EVs, DOX, or a mixture of both using UV or LIF detection. (B) Monitoring of the efficiency of DOX loading into EVs during incubation and sample purification with SEC. (C) Assessment of the stability of DOX–EV formulations stored at 4 °C and analyzed on the 1st, 2nd, 6th, 10th, and 14th day of storage. CE analyses were performed at 10 kV using UV (230 nm) or LIF (488/630 nm) detection and the BGE was composed of 50 mM Tris and 200 mM HEPES (pH 6.9). The temperature was set at 25 °C. The DOX concentration in the analyzed samples was equal to 10 µg mL^−1^.

A CE method was developed for studying DOX incorporation into EVs. For this purpose, the separation conditions were optimized to simultaneously separate DOX and EVs. This was not achievable with our previously published method because of the relatively high pH value of the BGE (pH 9.5), in which DOX was co-migrating with EVs in an anionic form and was not featuring fluorescence (Steć, Chodkowska, Kasprzyk-Pochopień, et al., 2023). For the protonation of the primary amino group of DOX, the pH of the BGE was decreased to 6.9 (the p*K*_a_ of DOX is 8.01). The ionic strength was adjusted with buffer compounds (Tris and HEPES) to counter-act DOX adsorption to the capillary wall and to preserve EVs dispersion stability (Dziomba et al., 2019). Representative electropherograms obtained with the developed method are presented in Figure 2(A). Under the optimized conditions, EVs generated similar signals to the ones observed earlier (Steć, Chodkowska, Kasprzyk-Pochopień, et al., 2023). Signals were poorly efficient (<20,000 plates m^−1^) and partially co-migrated with the electroosmotic flow (EOF) due to the low electrophoretic mobility of the vesicles. It is noteworthy that the DOX signal was not observed with a UV detector due to the low concentration of the drug (10 µg mL^−1^). However, LIF detection was sensitive enough to monitor the migration of DOX. The apparent mobility of DOX was greater than the EOF (positive polarity) which indicates that the substance was migrating in a cationic form. It should be emphasized that under the applied detection conditions (excitation: 488 nm; emission: 630 nm), native vesicles (before DOX incorporation) did not feature any fluorescence (Figure 2(A)). In turn, the fluorescence of DOX and EVs was observed after co-incubation which confirms the interaction between the drug and the vesicles.

The obtained formulations were characterized with NTA, cryo-TEM, and ELS. No significant differences in size distribution between the native and drug-loaded vesicles were observed (Figure 1(A)). DOX incorporation did not have any noticeable impact on the morphology of the vesicles (Figures S3 and S4). The zeta potentials of unmodified and DOX-loaded vesicles were also comparable (−25.0 ± 9 and −27.9 ± 8 mV, respectively; Figure S5).

**Figure 3.**
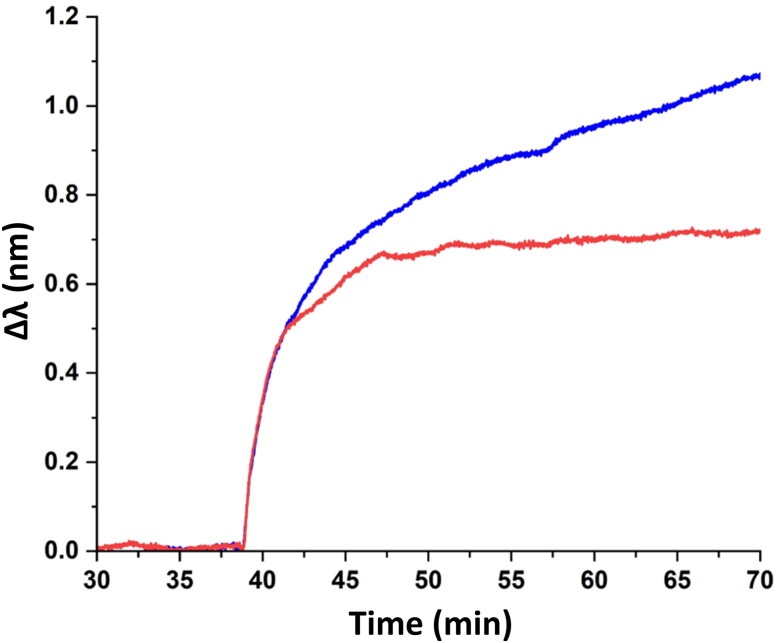
NPS of (blue line) native and (red line) DOX-loaded EVs.

**Figure 4.**
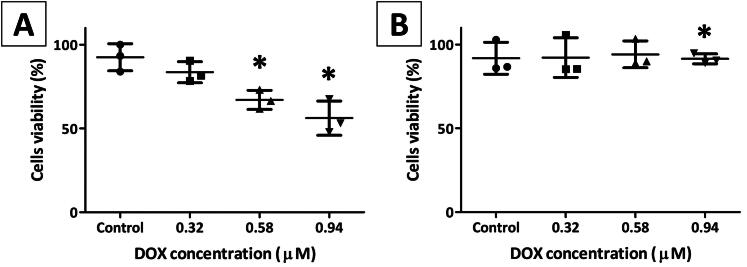
Viability of (A) HeLa and (B) HEK293T cells after 48 h of incubation with DOX-loaded EVs. The *x*-axis represents the total DOX concentration in cell culture reached with certain preparations used in the assay. Native, lemon-derived EVs in PBS solution were used as a control. Data are presented as the means of triplicate independent determinations with standard deviations (SDs). *Statistically significant results (*t*-test; Excel software; Microsoft Excel, Redmond, WA; *p* < .05).

Using the developed method, we were able to monitor the efficiency of DOX loading into plant-derived EVs. The impact of incubation time is shown in Figure 2(B). A significant increase in the fluorescence of EVs was observed after 24 h incubation with DOX as compared to 1 h. The vesicles also featured fluorescence after free DOX removal using SEC (Figure 2(B)) according to the procedure described in Section 2.3. The CE analysis of samples before and after SEC removal of free DOX enabled us to confirm that the applied methodology removed >99% of the free drug, at the same time providing high (>95%) recovery of EVs.

The CE method was also used for studying the formulation stability (Figure 2(C)). No significant differences were observed during the first few days after DOX incorporation. However, a noticeable EV peak splitting was detected on the 6th day after preparation of the formulations. At the same time, free DOX was also observed due to the drug leakage from the vesicles. Both phenomena (DOX leakage and EV signal splitting) were even more evident on the days 10th and 14th. Interestingly, neither the size distribution nor zeta potential of the analyzed particles changed during the storage period. The shift of migration time can be attributed to the presence of free DOX and its partial adsorption to the capillary wall.

### 3.3. NPS of native and DOX-loaded EVs

Nanoplasmonic sensing is a highly sensitive method based on the resonance of nanosensors (coinage metals like Au or Ag, in the present study Au coated with SiO_2_). The technique can detect minute changes in the refractive index of the immediate environment (2–20 nm) of the nanosensor. The change in the refractive index corresponds to various interactions factors, for example, drug binding affinity or any small changes in drug–nanoparticle complex. NPS studies on the interactions of drug molecules with nanoparticles such as liposomes, polymers, peptides, etc. are very scarce in the scientific literature. However, the interactions of different drugs with supported lipid bilayers have been reported recently (Jaikishan, Lavainne, Ravald, et al., 2024). Similarly, NPS studies on drug loaded EVs are nonexistent to the best of our knowledge. This work presents the novelty of applying NPS in comparing DOX-encapsulated EVS with pristine EVs obtained from citrus lemon. Considering the challenges and difficulties in acquiring and purifying EVs in bulk amount, NPS provides the edge over other techniques due to its sensitivity for very low concentration samples (Jaikishan, Lavainne, Wiedmer 2024). NPS is advantageous when it comes to sensitivity, expenses, high-throughput, or real-time interaction studies at nanoscale over other conventional techniques like mass spectrometry, UV spectroscopy, centrifugation, HPLC, or fluorescence-based methods. Additionally, drug incorporation into EVs in the present work was conducted by ‘encapsulation’ rather than by surface adsorption or chemical conjugation, which is beneficial regarding drug loading efficiency, ease of drug loading, drug stability, controlled release, reduced side effects and toxicity of the encapsulated drug, as well as controlled circulation time.

Plant-derived EVs without DOX as well as EVs loaded with the drug were successfully immobilized onto silicon dioxide (SiO_2_)-coated sensors, using standard protocols for the immobilization of liposomes (Witos et al., 2017). The Δ*λ* value for the original EVs was observed to be approximately 1.1 nm after 30 min and seemed to rise continuously (blue line in Figure 3). However, the Δ*λ* value of DOX-loaded EVs reached a maximum plateau value of 0.7 nm at about 15–20 min and remained constant (red line in Figure 3). The initial kinetics of the adsorption appeared to be almost similar for up to 5 min for both native and DOX-loaded EVs, suggesting a similar initial adsorption process. Native EVs are expected to have a slightly more rigid membrane than DOX-filled EVs. Studies have shown that a more rigid membrane structure of liposomes (composed of 1,2-dipalmitoyl-*sn*-glycero-3-phosphocholine, DPPC) results in higher Δ*λ* values as compared to fluid membrane composed of 1,2-dioleoyl-*sn*-glycero-3-phosphocholine (DOPC) under similar conditions (Oh et al., 2015). The greater fluidity of the hydrophobic core of the membrane was due to the presence of unsaturated acyl chains. DOX-loaded EVs are expected to have comparatively fluid membranes as reported earlier in studies on synthetic membranes (Alves et al., 2017) and plasma membranes (de Wolf et al., 1993; Peetla et al., 2010). Daunorubicin (a DOX analogue) has also been shown to destabilize non-lamellar membrane structures (Escribá et al., 1995). DOX has been shown to disrupt membrane structures, both at the interfacial region as well as in the hydrophobic core of the membrane (Alves et al., 2017). Therefore, another reason for the lower Δ*λ* value for EVs loaded with DOX (as compared to native EVs) could be the presence of DOX in the interfacial region of EVs and its interaction with charged phospholipids such as phosphatidylserines. Furthermore, DOX is positively charged at pH 7.4 and hence tends to interact with the headgroup regions of negatively charged phospholipids. Such an arrangement may lead to a lower phospholipid density on the sensor surface (Matyszewska et al., 2021), hindering the adsorption of DOX-loaded EVs on the SiO_2_-coated sensor surface, and thus leading to a lower Δ*λ* value.

### 3.4. Cytotoxicity assay and EVs modification

The DOX cytotoxicity to HEK293T and HeLa cell lines was determined using the CCK-8 kit. WST-8 reagent was reduced to formazan by NADH and NADPH which was generated by cellular dehydrogenase of living cells. The biochemical reaction caused a change in the color of the substrate which enabled the measurement of absorbance to assess the percentage of living cells. The IC_50_ of free DOX was determined to be 0.18 μM and 0.92 μM for HEK293T and HeLa, respectively. The isolated EVs, devoid of DOX, had no toxic effect on the cells (Figure 4).

Cytotoxicity of DOX-loaded EVs was assessed using three independently obtained preparations (according to the description in Sections 2.2 and 2.3). The total DOX concentration in each preparation was assessed by spectrophotometric measurement (Section 2.4) and was found to be 3.53, 6.42, and 10.36 µM. The protein content in each of these preparations was: 0.12, 0.46, and 0.44 mg mL^−1^, respectively. The average (±standard deviation) DOX loading efficiency was found to be 22.2 (±6.3) nM of DOX per µg of protein. Ten microliters of each preparation was mixed with 100 µL of cell suspension, resulting in 0.32, 0.58, and 0.94 µM of the final drug concentration, respectively. The free DOX content in each preparation was estimated by CE and was marginal compared to the incorporated DOX. DOX-loaded EVs inhibited cell proliferation and, consequently, caused cell death. A dose-dependent cell viability was observed for HeLa line (Figure 4(A)). The cytotoxic effect for nearly 50% of HeLa cells was achieved after 48 h of incubation with the highest DOX concentration (0.94 μM). This result is comparable with IC_50_ value determined for free DOX (0.92 μM). In turn, virtually no statistically significant differences in the viability of the HEK293T cell line were observed for the tested preparations (Figure 4(B)). The obtained results indicate that HEK293T cells were more sensitive than HeLa cells to free DOX. The incorporation of the drug into EVs had no apparent impact on the cytotoxicity of DOX against the HeLa cell line but significantly decreased the activity against HEK293T cells (at least fivefold).

### 3.5. DOX localization in the cell

**Figure 5.**
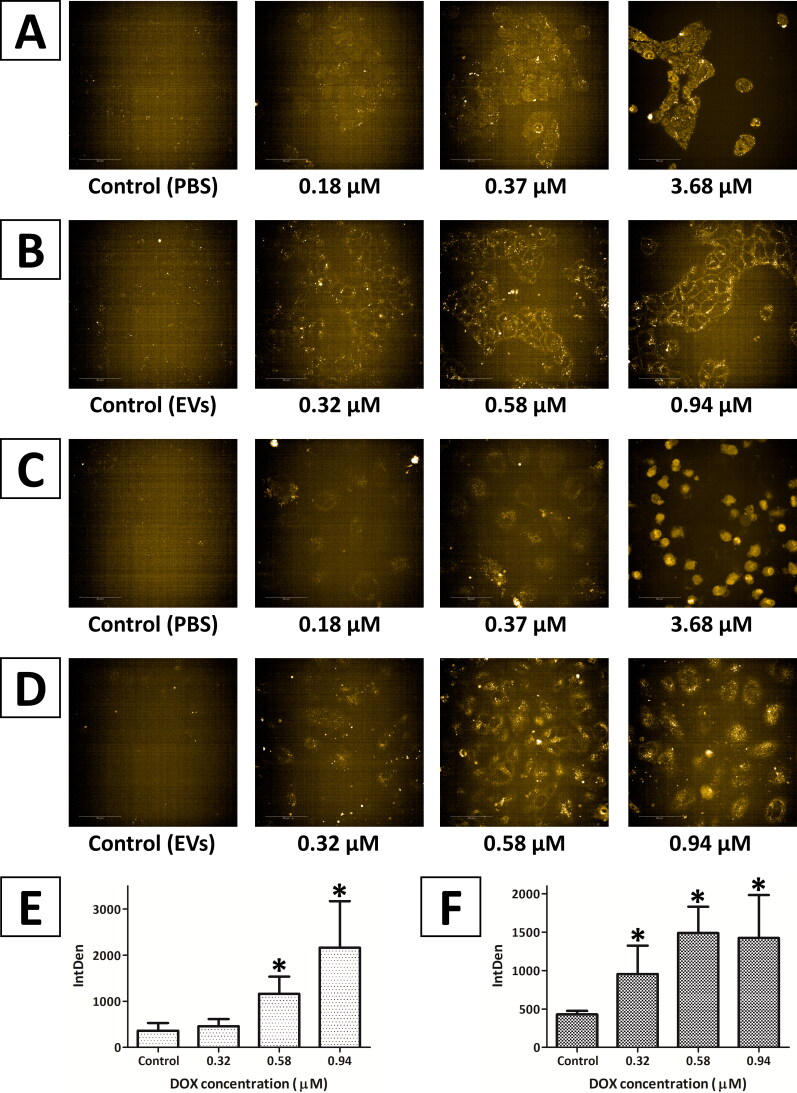
Confocal microscopy images of (A, B) HEK293T and (C, D) HeLa cells cultured in the presence of (A, C) free DOX and (B, D) DOX-loaded EVs for 48 h. Mean relative fluorescence intensity with standard deviation was calculated as single cells analysis of (E) HEK293T and (F) HeLa cells incubated with DOX-loaded EVs using ImageJ software. Controls: (A, C) PBS solution; (B, D) native EVs. *Statistically significant results (*p* < .05) for *n* = 15 (*t*-test; Excel software; Microsoft Excel, Redmond, WA).

The intracellular localization of DOX was visualized as yellow fluorescence using confocal microscopy (Figure 5). The obtained images show that after incubation of the cells with free DOX, the drug was located mainly in the cell nuclei and partially in the cytoplasm (Figure 5(A,C)). In the case of DOX-loaded EVs, the drug was mainly detected as yellow dots located in the cytoplasm (Figure 5(B,D)). The highest concentration of DOX caused changes in cell morphology and cell shrinkage due to cell death (Figure 5(A,C)).

The fluorescence intensity varied depending on the DOX concentration in the tested preparations. The analysis using ImageJ software (Bethesda, MD) indicated that the level of fluorescence (IntDen; Schneider et al., 2012) was consistent with the concentration of DOX in the preparations (Figure 5(E,F)).

## 4. Discussion

In recent years, EVs have emerged as a promising new class of drug nanocarriers (Elsharkasy et al., 2020). The costs and technical challenges related to the production of EVs from e.g. mesenchymal stem cells limit their industrial utility. Plant material is among the most important alternative sources of EVs for industrial applications (Paolini et al., 2022). Furthermore, plant-derived EVs are generally considered biocompatible, stable, and able to penetrate biological membranes without triggering an immunological response, which makes them a compelling alternative to liposomes (Wiklander et al., 2019).

The anticancer effect of *citrus*-derived EVs has already been demonstrated. *In vitro* studies involving EVs isolated from *Citrus limon* proved their ability to suppress the proliferation of diverse cancer cell lines which resulted in synergistic effect between vesicles and DOX (Raimondo et al., 2015). *C. limon*-derived EVs were also reported to induce cell cycle arrest in the S phase of gastric cancer cells and to trigger apoptosis (Yang et al., 2020). The anticancer effects of EVs on gastric cancer cells were mediated by the generation of reactive oxygen species. Furthermore, EVs were found safe and capable of persisting within the gastrointestinal tract.

The major side effect of DOX is its cardiotoxicity, which occurrence probability can be significantly reduced by the application of drug carriers (Chatterjee et al., 2010). The application of EVs as a smart delivery vehicle for DOX has already been tested including EVs isolated from bovine milk (Chen et al., 2021; Mukhopadhya et al., 2023), plant material (Niu et al., 2021), and eukaryotic cell line cultures (Martins-Marques et al., 2016; Schindler et al., 2019; Li et al., 2022; Patras et al., 2022; Chen et al., 2023; Mukhopadhya et al., 2023). In a few of the mentioned publications, co-incubation was found to be an efficient method for the drug incorporation (Li et al., 2022; Patras et al., 2022; Chen et al., 2023; Mukhopadhya et al., 2023), which was also confirmed in the presented study with the developed CE method.

It was observed that the EVs became fluorescent after their co-incubation with DOX, which indicates that there are interactions between the drug and the vesicles (Figure 2(A)). The fluorescence of the EVs was evident even after the removal of free DOX (Figure 2(B)). This observation implies that the DOX–EVs interaction was not transient, and the drug was absorbed by the vesicles. This conclusion is in line with the work of Chen et al. who reported that only a small dose of DOX is bound to the outer surface of vesicles while the majority of the drug is incorporated into EVs (Chen et al., 2023). On the other hand, significant differences between the native and drug-loaded EVs observed by NPS suggest that DOX is accumulated in the interfacial region of the carriers (Figure 3). Interestingly, drug loading caused no measurable differences in either electrophoretic mobility or zeta potential of the vesicles (Figure 2(A) and Figure S5), which demonstrates the superior sensitivity of NPS in this matter.

The great advantage of the presented CE method is its ability to investigate drug content in EVs even in the presence of free DOX (Figure 2(B)). This methodology can be used for studying the drug incorporation process and the stability of the formulation. We have demonstrated that the loading conditions greatly affect the fluorescence of EVs, which reflects the amount of incorporated DOX (Figure 2(B,C)). The method can also be used for the assessment of the efficiency of free DOX removal and to observe the drug leakage from the vesicles during storage. The latter aspect is especially important as, according to the regulatory guidance, formulations containing encapsulated drugs have to undergo additional stability assays including drug leakage evaluation (Food and Drug Administration, 2003, 2018).

Nevertheless, an implementation of the CE method into an industrial quality control laboratory will require method validation. A similar methodology has already been validated for the quality control of liposomal DOX formulations using CE (Kim & Wainer, 2010; Ansar et al., 2018; Jayaraj et al., 2024). Determination of DOX incorporated into vesicles might be more challenging because fluorescence is strongly dependent on the pH value and intermolecular interactions between the fluorophore and the biological components of the target (e.g. fluorescence quenching phenomenon). However, even without validation, the method is applicable for the optimization of the conditions for drug incorporation, rapid assessment of the formulation quality, as well as for estimating DOX leakage from the carrier. It should be emphasized that the presented approach is straightforward, does not require any additional sample preparation steps or free DOX removal before the analysis, which is a common practice in other types of experiments (Martins-Marques et al., 2016; Chen et al., 2021; Niu et al., 2021; Li et al., 2022; Mukhopadhya et al., 2023).

The study on HeLa and HEK293T cell lines revealed that EVs isolated from *Citrus limon* do not have cytotoxic effects and can be considered safe drug carriers. Furthermore, the tested cell lines displayed various susceptibilities to the free DOX and DOX-loaded EVs. HEK293T cells were more vulnerable to free DOX than HeLa cells. Interestingly, HeLa cells were more susceptible to DOX incorporated into EVs as compared to HEK293T cells. The activity of the DOX-loaded EVs on the cells led to an inhibition of cell proliferation and, subsequently, cell death over a longer time period as compared to free DOX. We observed that DOX-loaded EVs penetrate the cell membrane and localize in the cytoplasm (Figure 5). Then, DOX is slowly released from the EVs inside the cells, causing a cytotoxic effect. Based on this observation, we hypothesize that the EVs isolated from *Citrus limon* can be used as sustained-release drug carriers. Such a controlled drug release may reduce the adverse effects of some drugs such as DOX, help to control the intracellular concentration of the drug, and protect the labile drugs from, e.g. serum proteases. Moreover, the ability of the EVs to penetrate the cell membrane suggests that they can be used as carriers for drugs that are poorly soluble in water which targets cell organelles.

Cytotoxicity experiments showed that DOX concentrations in preparations obtained with the developed incorporation methodology were insufficient to determine IC_50_. Some attempts were made to increase the incorporation of DOX into the EVs. A further extension of the incubation time of EVs with DOX over 24 h was ineffective whereas increasing the drug concentration during co-incubation with the vesicles turned out to be impossible due to DOX precipitation (Yamada, 2020). Interestingly, DOX precipitation was neither observed in deionized water nor in PBS solution, even when relatively high concentrations of the substance were used (1–10 mg mL^−1^). Precipitation was only observed whenever the DOX solution was mixed with EVs dispersed in PBS solution, and the final concentration of DOX in such mixture exceeded 0.01 mg mL^−1^ (18.4 µM). It should be noted that DOX precipitation at such low concentrations is not easily recognizable. In our case, DOX precipitation was observed as a fine dispersion that underwent sedimentation after a few hours of the sample resting. Therefore, to increase the concentration of DOX in the preparation, higher concentrations of EVs during co-incubation should be considered in further attempts. This requires adapting the scale and methodology of EV isolation. Alternative solutions proposed in the literature include chemical modification of the carrier, which increases the uptake capacity of the vesicles (Niu et al., 2021), and active loading of the drug using an ion-gradient (Chen et al., 2021). Conjugation of the carrier with specific ligands for sensitization of the targeted cells to the drug-loaded carrier might also be taken into account.

## 5. Conclusions

The developed CE and NPS methods are promising tools for the analytical control of drug loading into EVs. Both approaches are complementary and provide exhaustive information on the quality of EV preparations. Despite the fact that under the experimental conditions the maximal achievable concentration of DOX was not sufficient to fully assess the cytotoxicity of drug-loaded EVs, their activity was shown to be dependent on the tested cell line. Further investigation of the drug release mechanism from vesicles might help to develop smart drug nanocarriers for medical applications in the future.

## Supplementary Material

Supplementary material.docx

## Data Availability

The data presented in this study are available on request from the corresponding author.
