# Supplementary material for: Incorporation of doxorubicin into plant-derived nanovesicles: process monitoring and activity assessment
Source: Drug Deliv. 2024 Dec 11;32(1):2439272. doi: 10.1080/10717544.2024.2439272 (PMC11639057; doi:10.1080/10717544.2024.2439272)
Supplement: Supplementary material.docx [file IDRD_A_2439272_SM5906.docx]

*Supplementary material*

**Incorporation of doxorubicin into plant-derived nanovesicles: process monitoring and activity assessment**

Aleksandra Steć^a*^, Monika Targońska^b*^, Shishir Jaikishan^c^, Rui Chen^c^, Piotr Mucha^d^, Grzegorz S. Czyrski^e^, Jacek Jasiecki^f^, Agata Płoska^g^, Andrea Heinz^e^, Susanne K. Wiedmer^c^, Leszek Kalinowski^g,h^, Krzysztof Waleron^f^, Bartosz Wielgomas^a^, Szymon Dziomba^a**^

^a^ Department of Toxicology, Faculty of Pharmacy, Medical University of Gdansk, 107 Hallera Street, 80-416 Gdansk, Poland

^b^ Department of Biology and Medical Genetics, Medical University of Gdansk, 1 Debinki Street, 80-211 Gdansk, Poland

^c^ Department of Chemistry, A.I. Virtasen aukio 1, 00014 University of Helsinki, Helsinki, Finland

^d^ Laboratory of Chemistry of Biologically Active Compounds, Faculty of Chemistry, University of Gdansk, 63 Wita Stwosza Street, 80-308 Gdansk, Poland

^e^ LEO Foundation Center for Cutaneous Drug Delivery, Department of Pharmacy, University of Copenhagen, 2100 Copenhagen, Denmark

^f^ Department of Pharmaceutical Microbiology, Faculty of Pharmacy, Medical University of Gdansk, 107 Hallera Street, 80-416 Gdansk, Poland

^g^ Department of Medical Laboratory Diagnostics—Fahrenheit Biobank BBMRI.pl, Faculty of Pharmacy, Medical University of Gdansk, 7 Debinki Street, 80-211 Gdansk, Poland

^h^ BioTechMed Centre, Department of Mechanics of Materials and Structures, Gdansk University of Technology, 11/12 Narutowicza Street, 80-233 Gdansk, Poland

* These authors contributed equally

** Email: szymon.dziomba@gumed.edu.pl





**Figure S1.** Original cryo-TEM image of the native vesicles in Figure 1C.





**Figure S2.** A zoom out cryo-TEM image of the native vesicles in Figure 1C.


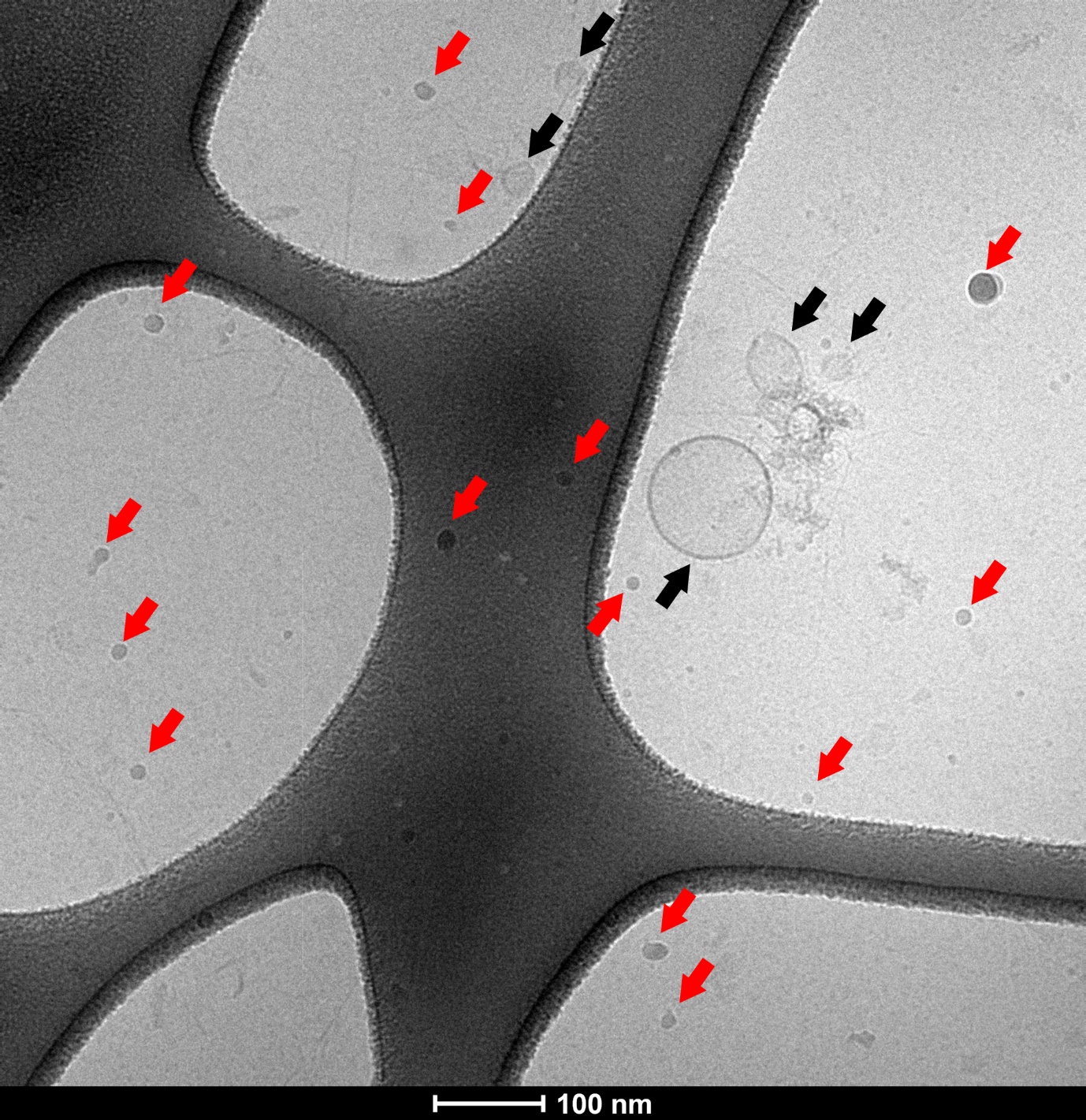


**Figure S3.** Cryo-TEM images of DOX-loaded plant-derived EVs. Black and red arrows indicate microvesicles and exosome-like plant vesicles, respectively.

**

**

**Figure S4.** A zoom out cryo-TEM image of the DOX-loaded plant-derived EVs.


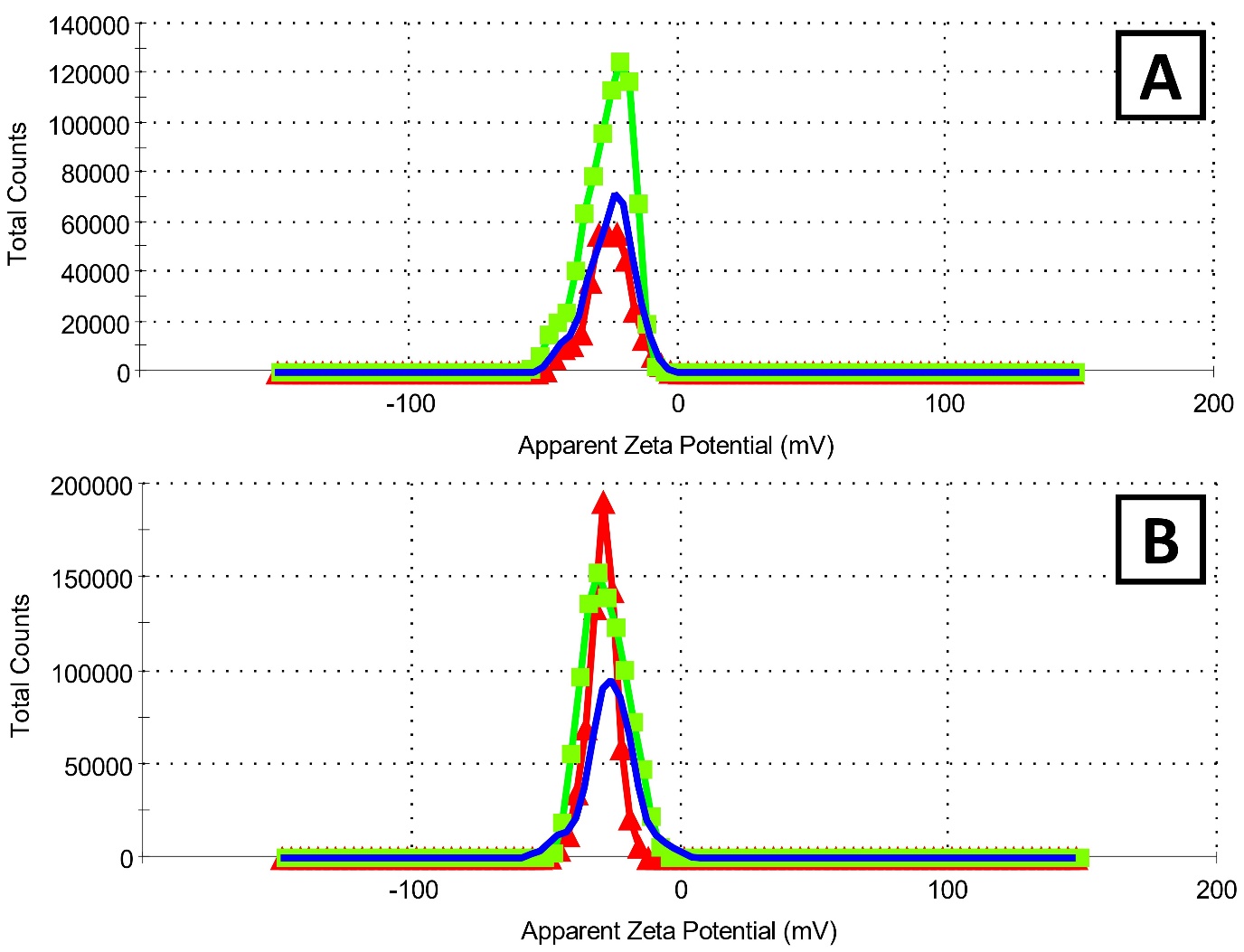


**Figure S5.** Zeta potential measurements of (A) native and (B) DOX-loaded plant-derived EVs. Each trace presented in the figure represents a single measurement of the same sample (the analysis was performed in triplicate).


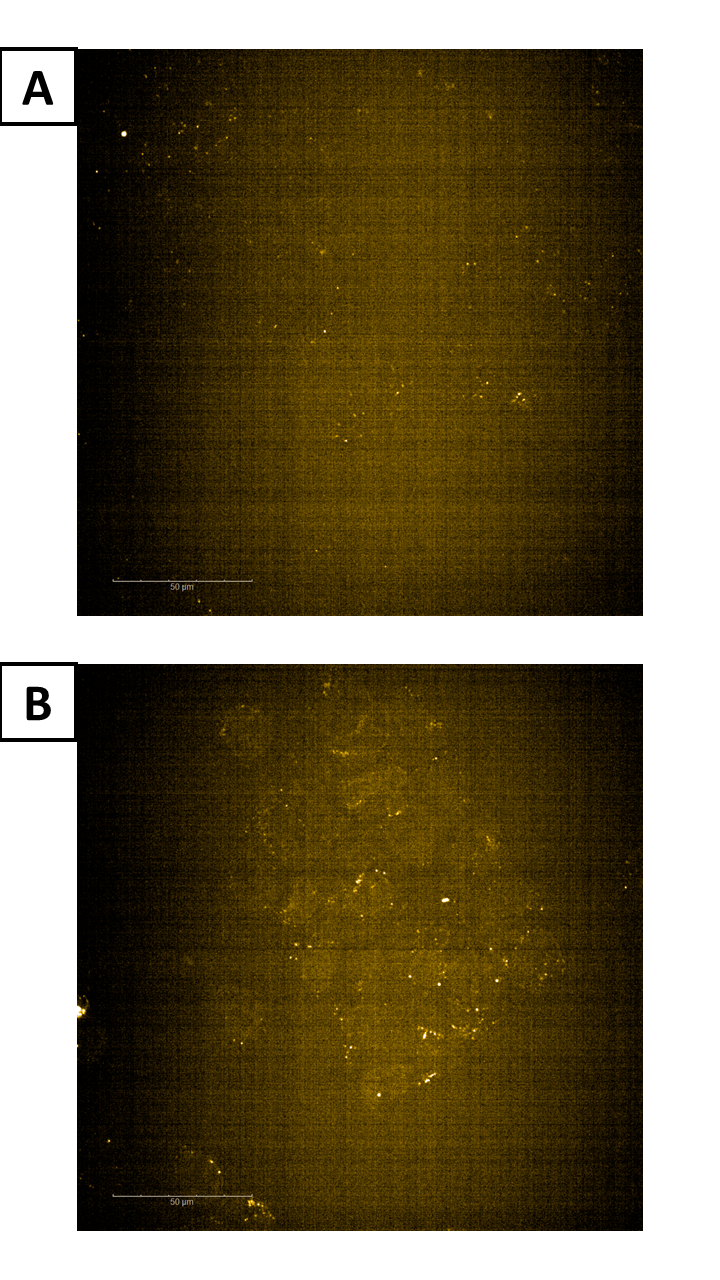


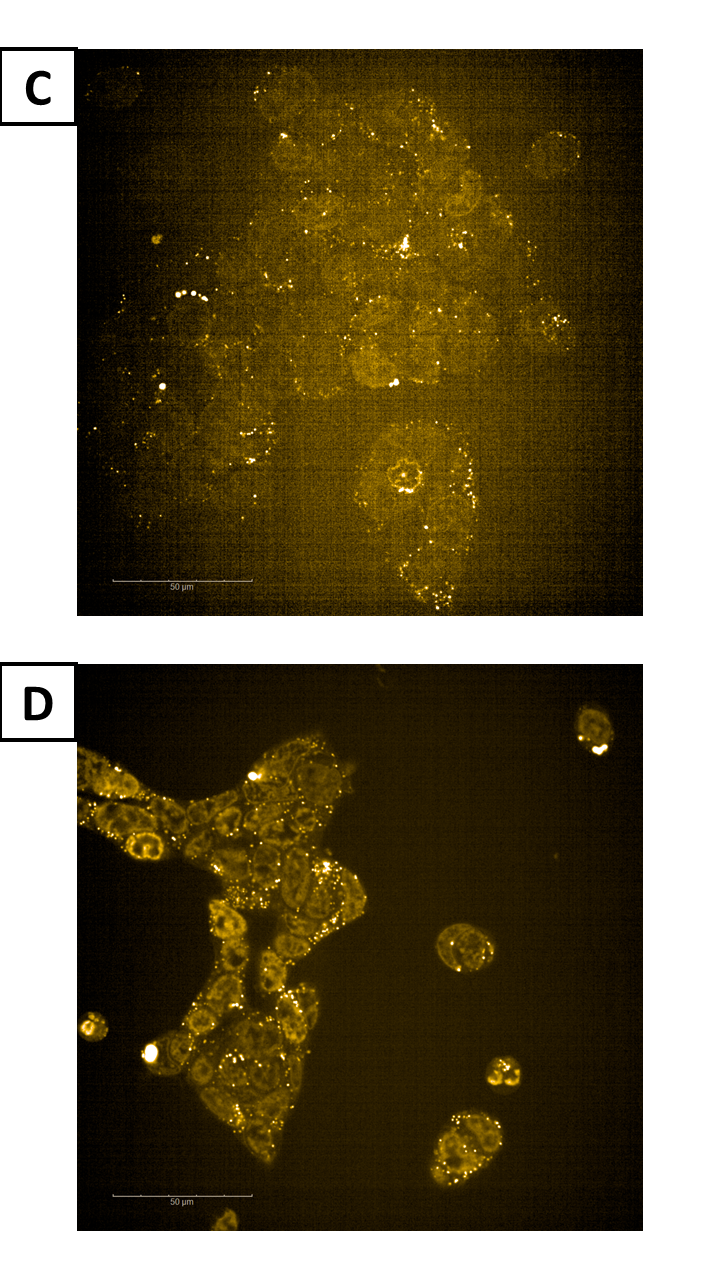


**Figure S6.** Confocal microscopy images of HEK293T cells cultured in the presence of free DOX for 48 h: (A) no DOX; (B) 0.18 μM DOX; (C) 0.37 μM DOX; (D) 3.68 μM DOX.


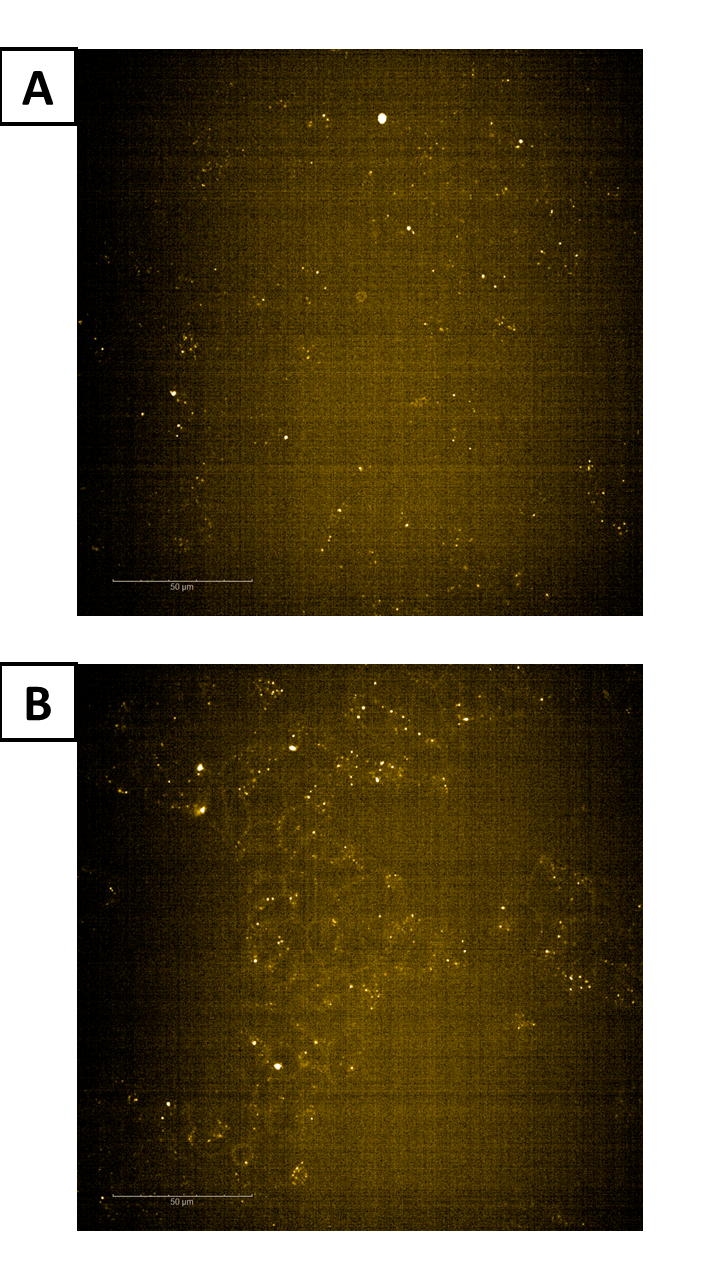


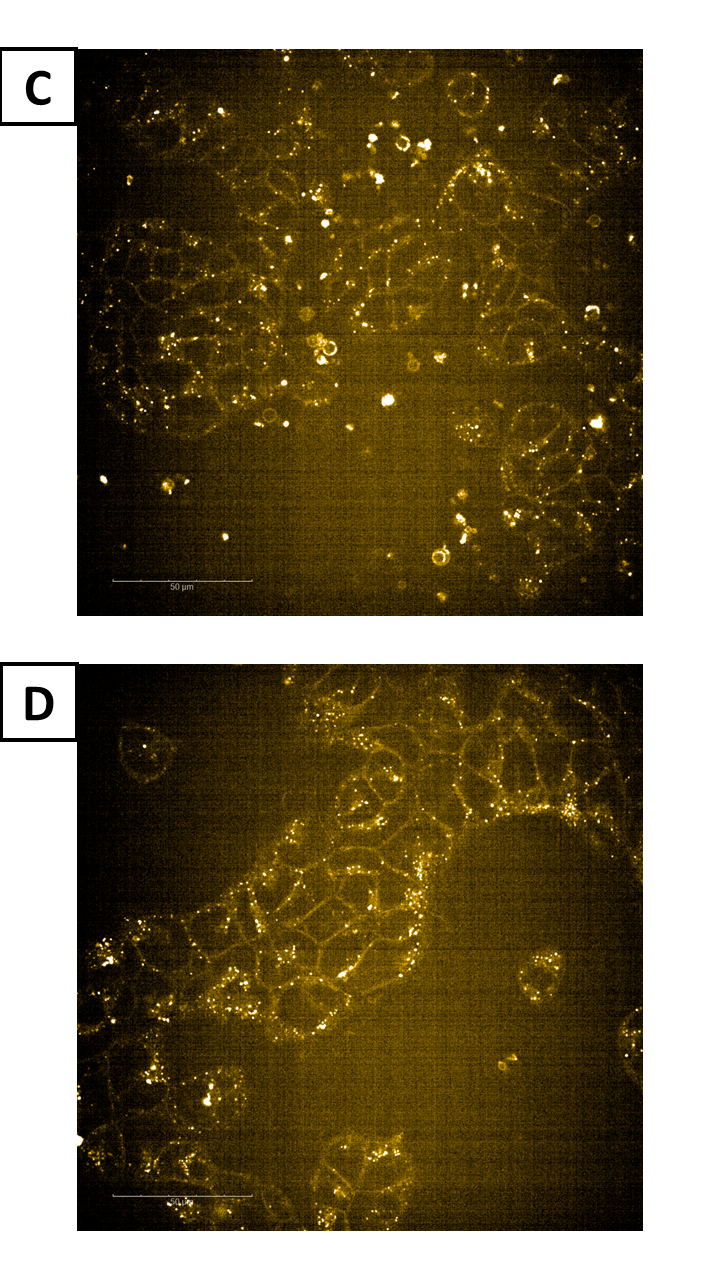


**Figure S7.** Confocal microscopy images of HEK293T cells cultured in the presence of DOX-loaded EVs for 48 h: (A) EVs not loaded with DOX; (B) 0.32 μM DOX; (C) 0.58 μM DOX; (D) 0.94 μM DOX.


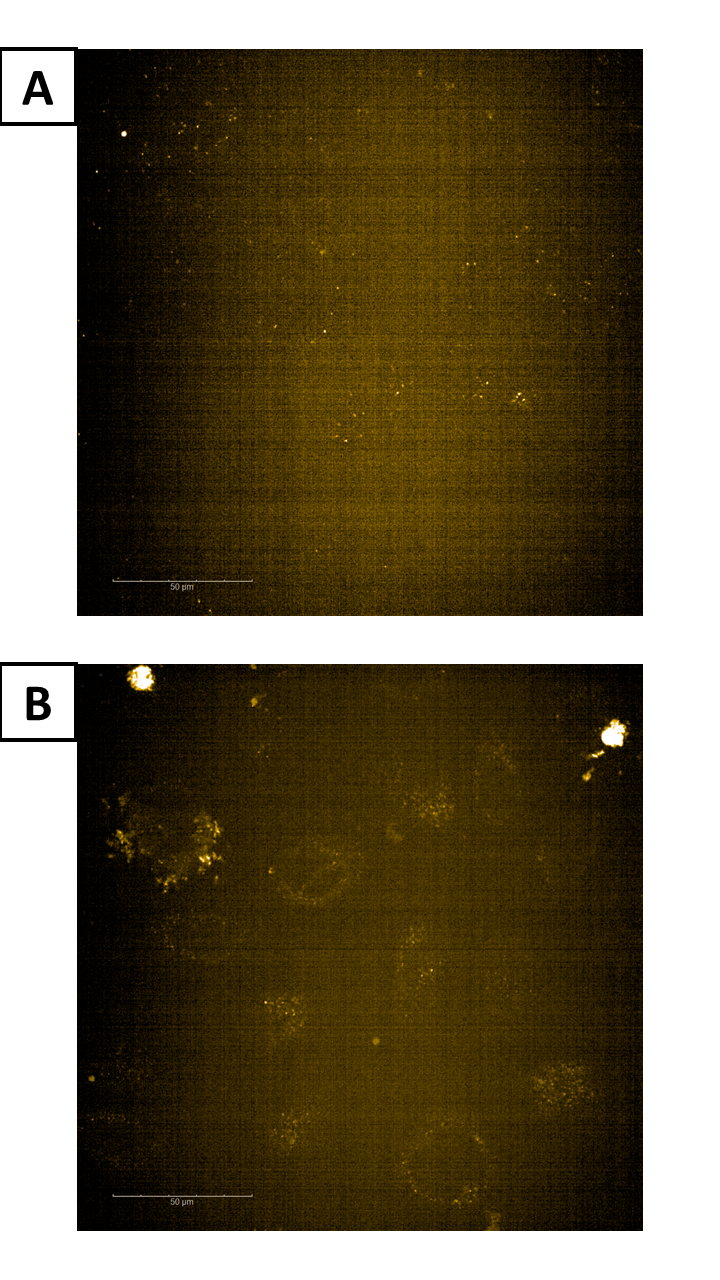


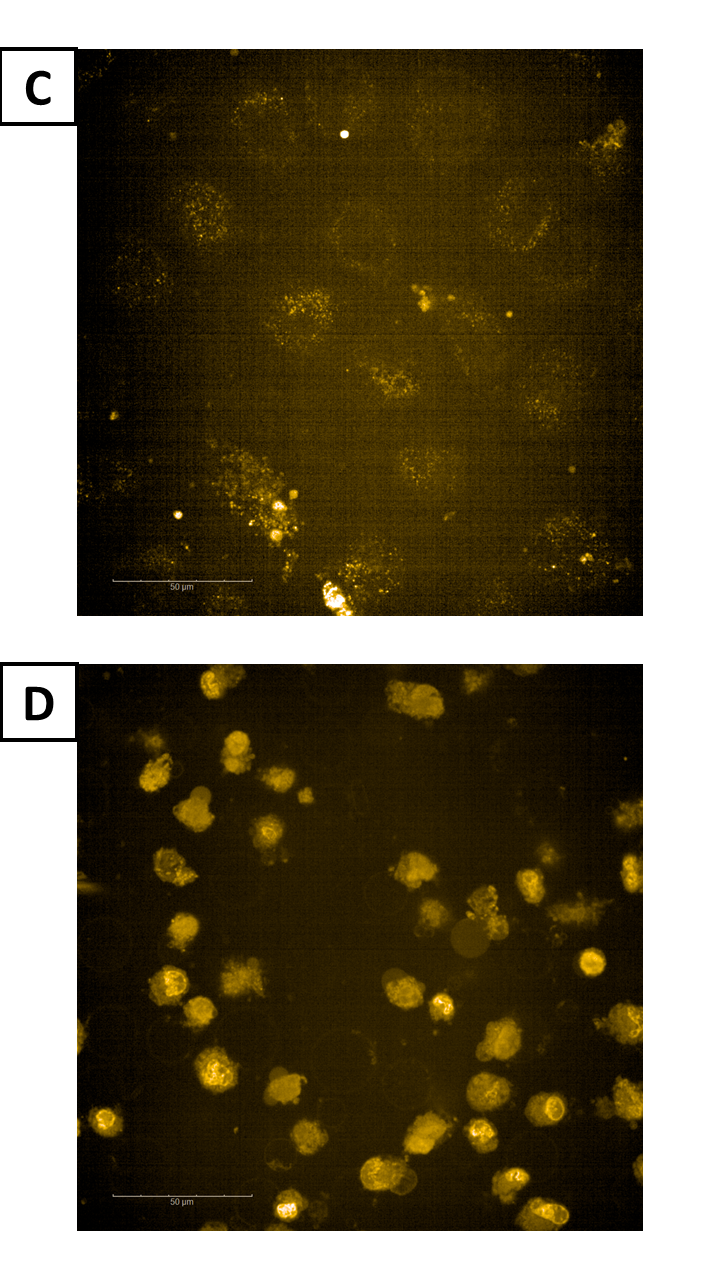


**Figure S8.** Confocal microscopy images of HeLa cells cultured in the presence of free DOX for 48 h: (A) no DOX; (B) 0.18 μM DOX; (C) 0.37 μM DOX; (D) 3.68 μM DOX.


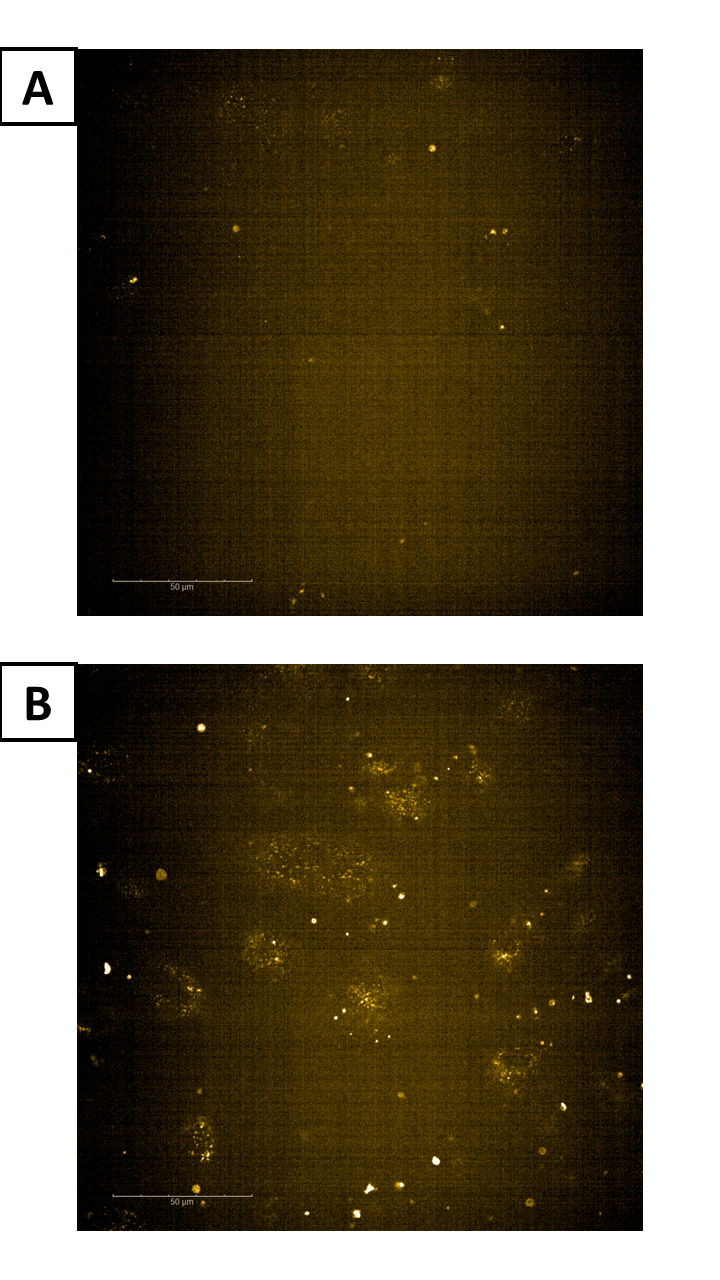


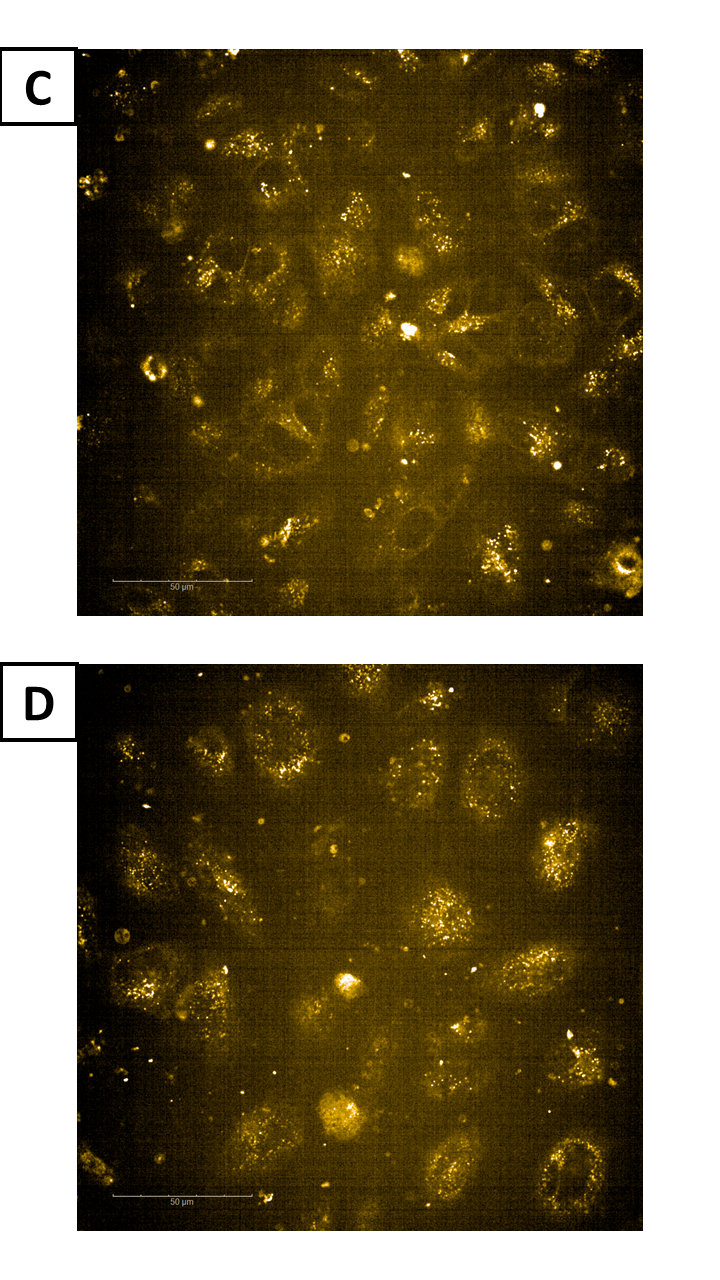


**Figure S9.** Confocal microscopy images of HeLa cells cultured in the presence of DOX-loaded EVs for 48 h: (A) EVs not loaded with DOX; (B) 0.32 μM DOX; (C) 0.58 μM DOX; (D) 0.94 μM DOX.
